# Localized and systemic bacterial infections in necrotizing pancreatitis submitted to surgical necrosectomy or percutaneous drainage of necrotic secretions

**DOI:** 10.1186/1471-2482-13-S2-S50

**Published:** 2013-10-08

**Authors:** Bruno Cacopardo, Marilia Rita Pinzone, Salvatore Berretta, Rossella Fisichella, Maria Di Vita, Guido Zanghì, Alessandro Cappellani, Giuseppe Nunnari, Antonio Zanghì

**Affiliations:** 1 Department of Clinical and Molecular Biomedicine, Division of Infectious Diseases, University of Catania, 95125 Catania, Italy; 2 Department of Surgery, General Surgery Unit, University of Catania, 95100 Catania, Italy

## Abstract

**Background:**

Infectious complications are observed in 40-70% of all patients with severe acute pancreatitis. Infections are associated with a significant increase in mortality rates.

**Methods:**

We evaluated the prevalence and characteristics of pancreatic and systemic infections in 46 patients with necrotizing pancreatitis submitted to surgical procedures during their hospital stay as well as the impact of such infectious complications on patient clinical outcome. Samples for microbiological cultures were taken at hospital admission from blood and bile and 2 days after invasive procedure from blood, drainage fluid, bile and necrotic tissues.

**Results:**

74% patients with necrotizing pancreatitis had a localized or systemic infection. At admission, 15% of subjects had positive blood cultures whereas 13% had evidence of bacterial growth from bile cultures. Two days after the invasive procedures for removal of necrotic materials and fluids, blood cultures became positive in 30% of patients in spite of antibiotic prophylaxis and bile cultures resulted positive in 22% of cases. Furthermore, bacterial growth from drainage fluids was found in 30% and from homogenized necrotic material in 44% of cases. As refers to bacterial isolates, all patients had a monomicrobial infection. Carbapenems were the drugs with the best sensitivity profile.

Mortality rate was significantly (p < 0.05) higher among patients with infection (17%) than subjects without infection (8%). Within the infected group, those subjects with evidence of systemic infection (positive blood cultures) developed more complications and demonstrated a higher (p < 0.05) mortality rate (28%) than those who had only a localized infection (10%).

**Conclusions:**

Infectious complications significantly increase mortality in patients with necrotizing pancreatitis. In addition, subjects with systemic infections developed more complications and demonstrated a higher mortality rate in comparison with those having a localized infection. In our study, the sensitivity pattern of the isolated microorganisms suggests to consider carbapenems as the best option for empirical treatment in patients with necrotizing pancreatitis who develop a clear-cut evidence of systemic or localized bacterial infection.

## Background

Infectious complications are observed in 40-70% of all patients with severe acute pancreatitis [1-3]. Mortality usually peaks within the first 7-10 days as a result of infectious complications, either limited to pancreatic necrotic areas or spread in the bloodstream [4,5].

Sterile pancreatic necrosis has a mortality rate of 20%, whereas it increases to more than 50% in the case of infected necrosis [6]. There is evidence that the involved bacteria originate from the gastrointestinal tract by transepithelial traslocation [7].

These patients are often given prophylactic antibiotics, although the use of this strategy may result in the development of an infection with resistant bacteria.

We conducted a study aimed at evaluating the prevalence and characteristics of pancreatic and systemic infections in patients with necrotizing pancreatitis submitted to surgical procedures during their hospital stay. We also evaluated the impact of such infectious complications on patient clinical outcome.

## Patients and methods

46 patients affected with acute necrotizing pancreatitis were consecutively enrolled among those admitted over a five-year period (2006-2011) at the Department of Surgery, University Hospital of Catania. The diagnosis of acute pancreatitis was based on clinical features, elevated serum amylase and/or lipase levels (more than 4-fold the upper reference limit) and evidence of pancreatic abnormalities on contrast-enhanced computed tomography (CECT) of the abdomen. The most common etiologies were gallstones (21 cases), alcohol (14 cases) and pancreotoxic drugs (8 cases), whereas 3 cases originated from abdominal traumas. The CT Severity Index [8] was 10 in 22 cases, 9 in 14 patients and 8 in the remaining 10 patients. All patients were closely monitored with adequate amounts of intravenous fluids and pain management. Supportive measures such as enteral nutrition and antibiotic prophylaxis were adopted in all cases. All 46 cases were managed invasively: 16 cases were treated by open surgical removal of necrotic areas, 15 cases underwent percutaneous catheter drainage of necrotic secretions, 15 cases received minimally invasive retroperitoneal necrosectomy. Seven of 46 patients (15%) died within 3 weeks from the onset of the disease.

Samples for microbiological cultures were taken at hospital admission from blood and bile and 2 days after invasive procedure from blood, drainage fluid, bile and necrotic tissues. Necrotic tissue samples from patients undergoing necrosectomy were homogenized immediately before culture.

The protocol for the study was approved by the Ethical Committee of our institution and written informed consent was obtained from all patients. The study protocol conformed to the ethical guidelines of the Declaration of Helsinki.

Statistical analysis was carried out using the statistical software package SPSS version 17.0 (SPSS, Chicago, Illinois, USA). A two-tailed P value of less than 0.05 was considered significant. All quantitative variables were expressed as mean ± standard deviation (SD). The chi-square test and the Fisher's exact test were adopted for statistical comparisons.

## Results

In our study population, mean age was 63 ± 17 years, 24 (52%) were males. Comprehensively, in 34 of 46 (74%) patients with necrotizing pancreatitis cultures demonstrated the presence of a localized or systemic infection.

In more detail, at hospital admission 7 of 46 patients (15%) already showed positive blood cultures whereas 6 (13%) had evidence of bacterial growth from bile cultures. Two days after the invasive procedures for removal of necrotic materials and fluids, blood cultures became positive in 14 of 46 patients (30%) in spite of antibiotic prophylaxis and bile cultures resulted positive in 10 patients (22%). Furthermore, bacterial growth from drainage fluids was found in 14 patients (30%) and from homogenized necrotic material in 20 (44%) cases. All those patients with positive bile or blood cultures also had infection of necrotic material.

As refers to bacterial isolates, all patients had a monomicrobial infection. Table 1 shows the cultured microorganisms with reference to the source sites. Table 2 shows the antibiotic resistance rates of the isolated strains.

**Table 1:** Number of isolated strains and site of isolation among 46 patients with necrotizing pancreatitis

	N. isolated strains			
	**Blood**	**Bile**	**Drainage**	**Necrotic material**

**Gram-negative bacteria**				

Escherichia *coli*	4	4	7	10

Pseudomonas *aeruginosa*	2	2	1	3

Klebsiella *pneumoniae*	2	2	1	-

Proteus *mirabilis*	1	2	1	1

Acinetobacter *baumannii*	-	-	-	1

**Gram-positive bacteria**				

Coagulase negative *Staphylococci *(CNS)	6	2	1	1

Methicillin-resistant Staphylococcus *aureus (MRSA)*	1	-	1	2

Enterococcus *faecium*	3	2	1	1

Enterococcus *faecalis*	2	2	1	1

**Table 2:** Antibiotic resistance rates (%) of all the isolated bacteria among 46 patients with necrotizing pancreatitis

	E. coli	P. aeruginosa	K. pneumoniae	P. mirabilis	A. baumannii	E. faecium/faecalis	CNS	MRSA
Amoxi/Clavulanate	22	48	60	32	100	100	50	100

Amikacin	10	20	12	18	88	44	58	100

Ampi/Sulbactam	22	48	60	36	100	40	68	100

Cefotaxime	54	68	72	44	100	42	36	92

Ceftazidime	32	38	38	20	88	100	78	100

Ceftriaxone	40	60	44	42	100	100	56	92

Ciprofloxacin	24	82	62	26	92	42	22	78

**Imipenem**	**2**	**12**	**8**	**0**	**24**	**-**	**-**	**-**

Levofloxacin	24	82	52	16	100	40	20	56

**Meropenem**	**0**	**6**	**2**	**0**	**16**	**-**	**-**	**-**

Piperacillin/Tazobactam	22	48	26	16	58	90	40	100

Linezolid	-	-	-	-	-	2	0	2

Vancomycin	-	-	-	-	-	2	0	2

Rifampicin	80	100	68	88	2	2	0	68

Cotrimoxazole	32	100	40	38	100	100	0	50

MRSA: Methicillin-resistant Staphylococcus aureus; CNS: Coagulase negative Staphylococci

As refers to the clinical outcome, mortality rate was significantly (p < 0.05) higher among patients with infection (N = 34) than subjects without infection (N = 12): 6 (17%) vs. 1 (8%), respectively. Within the infected group, those 14 subjects with evidence of systemic infection (positive blood cultures) developed more complications (Table 3) and demonstrated a higher (p < 0.05) mortality rate in comparison with those who had only a localized infection (limited to necrotic material, bile or drainage fluids): 4 (28%) vs. 2 (10%), respectively.

**Table 3:** Complications developed among patients with evidence of systemic or localized infection

	Bloodstream infection N = 14	Infection of necrotic tissue/bile/drainage fluids N = 20
Pleural effusion	4 (28%)	2 (10%)

Pericardial effusion	3 (21%)	1 (5%)

Pneumonia	3 (21%)	0

Cerebral abscess	1 (7%)	0

Septic shock	3 (21%)	1 (5%)

DIC	1 (7%)	0

DIC: Disseminated Intravascular Coagulation

## Discussion

In the present study, we examined pancreatic and systemic infections in patients with necrotizing pancreatitis. In keeping with previous studies, the prevalence of infections in this setting usually correlates with the extent of pancreatic necrosis [9,10].

In our experience, comprehensive rate of infection was 74%, with an overall prevalence of bloodstream infections of 30% and a rate of localized infections within pancreatic necrotic areas exceeding 40%. Noor et al. [11] observed pancreatic infections in 37.3% of patients and extrapancreatic infections in 62.7% of patients with severe acute pancreatitis.

In a study by Garg et al. [12], extrapancreatic bacterial infections were found in 31.7% of 63 patients. Bourgaux et al. [13] reported extrapancreatic infections in 25% of their patients. The most common sites of infection were the peritoneal fluid (26.8%) and blood (24.4%). Finally, in a recent study by Besselink et al. [14], bacteremia was reported only in 13.4% of the enrolled cases.

Similarly to our data, in patients with pancreatic infections monomicrobial infections were reported to be more common than polymicrobial ones [11].

The most common mechanism for early infection in acute necrotizing pancreatitis involves bacterial translocation from the gut. This could explain the high frequency of pancreatic and systemic infections by Escherichia *coli *we found in our study. Recently, Su et al. [15] described Escherichia *coli *to be the most common microbe responsible for infection in patients with severe acute pancreatitis.

Infections with Gram-positive organisms could occur later due to nosocomial bloodstream spread [11]. Actually, a progressive shift from Gram-negative to Gram-positive organisms might occur either associated to the increasing length of hospital stay or related to the administration of prophylactic antibiotics mainly targeting Gram-negative bacteria [14].

Previous studies emphasized the role of pancreatic infections as unfavorable predictors of patient clinical outcome. Noor et al. [11] reported mortality to be significantly higher in patients with pancreatic infections. Also, in the study by Besselink et al. [14] patients with infected pancreatic necrotic areas had a higher mortality rate. Differently from them, our study showed that bacteremic infections throughout the course of necrotizing pancreatitis worsened the outcome of the disease much more than localized infections either in terms of development of complications or in terms of crude mortality rate. Actually, in the study by Besselink et al. [14] the highest mortality rates were found in those cases with concomitant pancreatic and bacteremic infection.

The results of the sensitivity pattern of the isolated microorganisms suggest that carbapenems should be evaluated for empirical treatment in those patients with necrotizing pancreatitis who develop a clear-cut evidence of systemic or localized bacterial infection.

## Competing interests

The authors declare that they have no competing interests.

## Authors' contributions

BC: conception and design, interpretation of data, statistical analysis, critical revision, given final approval of the version to be published. MRP: acquisition of data, drafting the manuscript, statistical analysis, given final approval of the version to be published. SB: acquisition of data, given final approval of the version to be published. RF: acquisition of data, given final approval of the version to be published. MDV: acquisition of data, given final approval of the version to be published. GZ: acquisition of data, given final approval of the version to be published. AC: acquisition of data, given final approval of the version to be published. GN: acquisition of data, drafting the manuscript, given final approval of the version to be published. AZ: interpretation of data, critical revision, given final approval of the version to be published.

## Authors' information

BC: Associate Professor of Infectious Diseases at University of Catania

MRP: Resident in Infectious Diseases at University of Catania

SB: Full Professor of Surgery at University of Catania

RF: Resident in Surgery at University of Catania

MDV: Assistant Professor of Surgery at University of Catania

GZ: Associate Professor of Surgery at University of Catania

AC: Associate Professor of Surgery at University of Catania

GN: Assistant Professor of Infectious Diseases at University of Catania

AZ: Associate Professor of Surgery at University of Catania

## Declarations

Publication of this article was funded by the Department of Clinical and Molecular Biomedicine, University of Catania.

This article has been published as part of *BMC Surgery *Volume 13 Supplement 2, 2013: Proceedings from the 26th National Congress of the Italian Society of Geriatric Surgery. The full contents of the supplement are available online at http://www.biomedcentral.com/bmcsurg/supplements/13/S2
